# TB Hackathon: Development and Comparison of Five Models to Predict Subnational Tuberculosis Prevalence in Pakistan

**DOI:** 10.3390/tropicalmed7010013

**Published:** 2022-01-17

**Authors:** Sandra Alba, Ente Rood, Fulvia Mecatti, Jennifer M. Ross, Peter J. Dodd, Stewart Chang, Matthys Potgieter, Gaia Bertarelli, Nathaniel J. Henry, Kate E. LeGrand, William Trouleau, Debebe Shaweno, Peter MacPherson, Zhi Zhen Qin, Christina Mergenthaler, Federica Giardina, Ellen-Wien Augustijn, Aurangzaib Quadir Baloch, Abdullah Latif

**Affiliations:** 1 KIT Royal Tropical Institute, 1092 AD Amsterdam, The Netherlands; e.rood@kit.nl (E.R.); c.mergenthaler@kit.nl (C.M.); 2 Department of Sociology and Social Sciences, University of Milano Bicocca, 20126 Milan, Italy; fulvia.mecatti@unimib.it; 3 Departments of Global Health and Medicine, University of Washington, Seattle, WA 98195, USA; jross3@uw.edu; 4 School of Health and Related Research, University of Sheffield, Sheffield S1 4DA, UK; p.j.dodd@sheffield.ac.uk (P.J.D.); d.shaweno@sheffield.ac.uk (D.S.); 5 Institute for Disease Modeling, Seattle, WA 98109, USA; stchang@idmod.org; 6 Epcon, 2000 Antwerp, Belgium; thys@epcon.ai; 7 Sant’Anna School of Advanced Studies, 56127 Pisa, Italy; gaia.bertarelli@santannapisa.it; 8 Big Data Institute, Nuffield Department of Medicine, University of Oxford, Oxford OX3 7LF, UK; nathaniel.henry@ndm.ox.ac.uk; 9 Institute for Health Metrics and Evaluation, University of Washington, Seattle, WA 98109, USA; kel15@uw.edu; 10 École Polytechnique Fédérale de Lausanne, 1015 Lausanne, Switzerland; William.trouleau@epfl.ch; 11 Malawi-Liverpool-Wellcome Trust Clinical Research Programme, Queen Elizabeth Central Hospital, Blantyre 312225, Malawi; Peter.MacPherson@lstmed.ac.uk; 12 Department of Clinical Sciences, Liverpool School of Tropical Medicine, Liverpool L3 5QA, UK; 13 Clinical Research Department, London School of Hygiene and Tropical Medicine, London WC1E 7HT, UK; 14 Stop TB Partnership, 1218 Geneva, Switzerland; zhizhenq@stoptb.org; 15 Department of Biostatistics, Radboud University Medical Centre, 6525 GA Nijmegen, The Netherlands; federica.giardina@radboudumc.nl; 16 Faculty of Geo-Information Science and Earth Observation (ITC), University of Twente, 7514 AE Enschede, The Netherlands; p.w.m.augustijn@utwente.nl; 17 Pakistan National Tuberculosis Control Programme, Islamabad 44000, Pakistan; aurangzaib.quadir@gmail.com (A.Q.B.); abdullah.latif@gmail.com (A.L.)

**Keywords:** small area estimation, tuberculosis burden, predictive modelling, subnational prevalence, spatial epidemiology, forecasting

## Abstract

Pakistan’s national tuberculosis control programme (NTP) is among the many programmes worldwide that value the importance of subnational tuberculosis (TB) burden estimates to support disease control efforts, but do not have reliable estimates. A hackathon was thus organised to solicit the development and comparison of several models for small area estimation of TB. The TB hackathon was launched in April 2019. Participating teams were requested to produce district-level estimates of bacteriologically positive TB prevalence among adults (over 15 years of age) for 2018. The NTP provided case-based data from their 2010–2011 TB prevalence survey, along with data relating to TB screening, testing and treatment for the period between 2010–2011 and 2018. Five teams submitted district-level TB prevalence estimates, methodological details and programming code. Although the geographical distribution of TB prevalence varied considerably across models, we identified several districts with consistently low notification-to-prevalence ratios. The hackathon highlighted the challenges of generating granular spatiotemporal TB prevalence forecasts based on a cross-sectional prevalence survey data and other data sources. Nevertheless, it provided a range of approaches to subnational disease modelling. The NTP’s use and plans for these outputs shows that, limitations notwithstanding, they can be valuable for programme planning.

## 1. Introduction

There is increasing demand for tuberculosis (TB) estimates at subnational level to inform TB programme planning in low and middle-income countries [1] Indeed, there is substantial geographical heterogeneity in TB prevalence in high TB burden countries. Subnational estimates are therefore considered valuable by national TB control programmes (NTP) to optimise resource allocation.

Case notifications are the main source of subnational TB data, but even in countries with good health coverage, these data may not reflect patterns in disease burden [1]. Reasons include: (1) cases from one reporting area may be diagnosed in neighbouring areas due to better access and quality of care (or people’s perception thereof); (2) even in countries where TB is a notifiable disease some service providers may not report through the national reporting system (e.g., private sector providers) [1]. Issues with subnational TB burden based on notifications are further exacerbated in contexts where access to care and reporting vary geographically.

National population-based prevalence surveys provide a direct measurement of the burden of disease. They are considered the gold standard in estimating the prevalence of TB, but they are typically not powered to provide subnational estimates of TB. Most TB prevalence surveys only allow the generation of reasonably precise estimates of TB prevalence at the national level in a small number of strata (e.g., two to three geographical regions) [1].

A number of approaches to produce sub-national estimates of TB burden have been proposed, but validating the accuracy of model predictions remains challenging. Indeed, these estimation methods are typically implemented in settings where the information need is greatest, which is almost always where there is no accurate empirical data against which to evaluate the validity of model predictions [2,3]. Notable examples of subnational TB burden estimation include the SUBsET model which aims to estimate subnational incidence of TB in Indonesia [4], Bayesian models to estimate subnational TB mortality [5] and incidence [6] in Brazil or TB prevalence in Cambodia [7]. Alternative approaches to subnational TB estimation include indirect methods to estimate sub-national notification gaps in Bangladesh [8], Nepal and Pakistan [9]. While the cited approaches implemented various statistical methods of validation, the extent to which predictions accurately reflect real burden often remains uncertain. To the best of our knowledge, there have been no attempts to apply more than one modelling approach in the same setting, meaning that cross-validation of approaches has not been attempted.

Pakistan’s NTP is among the many programmes worldwide who value the importance of subnational estimates of TB burden but do not have reliable estimates to support the TB response. Pakistan is a very diverse country with a population of 217 million in four provinces and three regions. TB is a major public health concern, with an estimated 570,000 new cases in 2019 and 43,900 deaths attributable to the disease [10]. Pakistan was among the eight countries that accounted for two thirds of the total global number of incident cases worldwide in 2019 [10]. Moreover, under-diagnosis and under-reporting of cases are considered key barriers to ending TB. Pakistan is among the five countries which accounted for more than half of the people with TB who are missed globally, as measured by the gap between the number of incident cases and the number of people notified on TB treatment in 2019 [10]. This is partly attributed to under-reporting by public and private providers who operate outside the NTP [11].

Against this backdrop, the Pakistan NTP partnered with epidemiologists at KIT Royal Tropical Institute to launch a virtual hackathon for the estimation of the subnational TB burden in Pakistan. Hackathons are problem solving events where participants collaborate intensively to develop a proposed solution for a specific issue within a short period of time. Hackathons originated in the field of software development and are increasingly proposed as a problem solving model in health. To date, most hackathons in global health have been conducted to develop new medical technologies [12,13] and especially solutions relying on mobile and wireless devices [14,15]. Examples also include the development of new analytical [16,17] methods and fostering multi-cultural dialogue [18]. One of hackathons’ most appealing features is the potential for greater innovation due to the heterogeneity of participants’ backgrounds and the encouragement of collaborations across institutions [16,19].

The Pakistan TB hackathon aimed to bring together various research groups interested in TB modelling to collaborate on a joint modelling exercise of the subnational TB burden in Pakistan in 2018. The hackathon’s objectives were two-fold: (1) to compare and cross-validate different models for small area estimation of TB in Pakistan; (2) to provide Pakistan NTP with data to tailor their efforts to ending TB to different sub-national contexts.

## 2. Methods

The TB hackathon was a virtual event which did not require people to be physically present in the same space at the same time. It was launched in April 2019 by means of announcements on the KIT website, on social media and in mailings lists. Participants were offered three months and the same set of data (Table 1) to develop their own models to estimate sub-national TB burden.

Participating teams were requested to produce district-level estimates of bacteriologically positive TB prevalence among adults (over 15 years of age) for 2018. The choice of 2018 as prediction year was the result of a compromise between the information needs of the NTP (who needed the most recent possible estimate for programme planning) and what was considered reasonable within the scope of available data for modelling (with auxiliary data for modelling expected to be available up until 2018 at the latest).

Nine teams applied for and signed data sharing agreements with the Pakistan NTP. Participating teams developed their models between mid-May and mid-September 2019. Five teams submitted district-level estimates for the whole country by the September 2019 deadline. Submitted models were appraised and compared by an evaluation panel comprising global TB and statistical experts including representatives from Pakistan’s NTP and the World Health Organisation. Feedback from the evaluation panel was provided to the modellers.

### 2.1. Data Sources

Table 1 provides a complete overview of the data provided to hackathon modellers. Participants were invited to use the data provided, and any other publicly available data, for their model.

**Figure 1:**
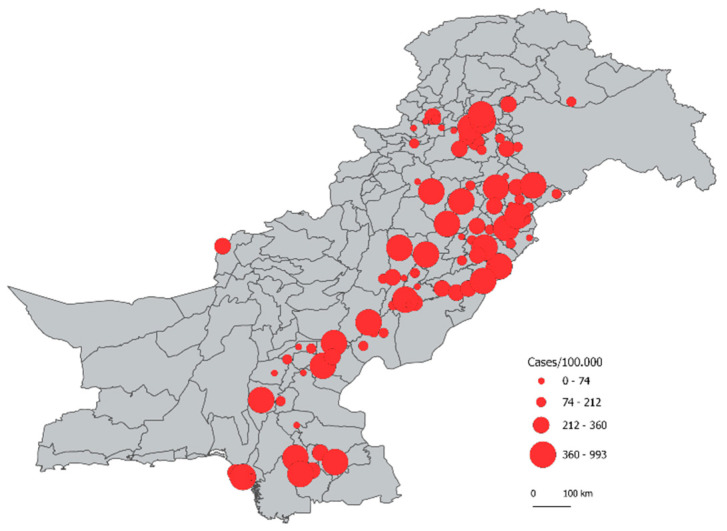
Proportion of TB bacteriologically positive people (out of all tested) by cluster in Pakistan in 2010–2011 prevalence survey [21].

The Pakistan NTP provided case-based data for their 2010–2011 TB prevalence survey data. The national TB prevalence survey was a nationwide cross-sectional survey with multistage cluster sampling conducted in 95 clusters from 68 districts [20]. The clusters corresponded to *tehsils* (sub-districts) and were selected using sampling proportional to the estimated *tehsil* population size in 2010 projected from 1998 census data [20,21]. The Federally Administered Tribal Areas, district Dera Bugti in Balochistan, and 17 *tehsils*, of Khyber Pakhtunkhwa, were excluded from the survey due to serious security threats. Combined, these excluded areas account for 6.4% of Pakistan’s population. A total of 105,913 adults (≥15 years of age) participated in the survey, of whom 10,471 (9.9%) were eligible for sputum examination [20]. Of these, 8521 (81.4%) submitted at least one specimen for sputum examination. The proportion of TB bacteriologically positive people (out of all tested) by cluster according to the 2010–2011 prevalence survey is shown in Figure 1 [20]. (These do not take into account the Pakistan TB prevalence survey report adjustment for missing TB results among participants eligible for smear examination or non-participation.).

The Pakistan NTP also shared data relating to TB testing and treatment, in both the private and public sectors, for the period between 2010 and 2018 (Table 1) to fill the gap between the year of the TB prevalence survey (2010–2011) and the year for which predictions were requested (2018). This was complemented by further data on HIV registrations as well as screening and testing for HIV in TB patients (since people living with HIV are more likely than others to develop TB disease). Participants were also directed to the 2017 census provisional province-wise population counts by sex and rural or urban location [22] and overall population counts by *tehsil* [23] (which were openly available online at the time). To ensure geographical consistency across all model predictions, participants were provided with the latest district shapefiles obtained from GADM (gadm.org). In April 2019, the GADM versioning included 143 out of the 146 districts which existed up to the 2017 census (in addition to the frequent redrawing of district boundaries in Pakistan, there was a major overhaul as part of the 2017 census, leading to the current 156 districts).

### 2.2. Comparison of Models and Predictions

In the absence of an empirical ground truth to evaluate the predictions (e.g., a 2018 subnational prevalence survey), we first compared the models and then appraised the quality of predictions using maps and a series of bespoke data quality indicators.

To compare models, we described the modelling building approaches (modelling and inferential frameworks, covariance structures, selection of final model, post-modelling processing of predictions) and strategies for variable selection (choice of outcome and predictor variables, variable processing, lowest level of spatial aggregation).

To compare predictions, we first mapped each model’s 2018 district estimate on common scales. We then calculated summary statistics and data quality indicators of completeness, pseudo-accuracy, precision, cross-validity and credibility. We assessed completeness as the proportion of Pakistan’s districts for which predictions were available (out of 143). For pseudo-accuracy, hackathon modellers were requested to perform leave-one-out-cross-validation and provide the R^2^ comparing actual and predicted 2010–2011 cluster-level prevalence point estimates (since there was no prevalence survey or other empirical data in 2018 this calculation could not be made for the 2018 predictions). For precision, we calculated an approximated coefficient of variation as the difference between the upper limit and lower limit of 95% CI divided by the point estimate. Cross-validity was assessed by comparing model predictions using scatter plots and Pearson’s correlation coefficient. We also produced a number prediction plots (including histograms, precision plots and pairwise correlations) to support this comparison of predictions.

The indicator of credibility differs from all others as it is based on expert opinion. We presented anonymised maps of each model’s 2018 district estimates to four Pakistan TB experts (from the Pakistan NTP) and asked them to grade the estimates on a scale from 1 to 10 based on how credible they deemed the estimates, based on their knowledge of the TB epidemic in their country. We presented individual grades and summary statistics (mean) and calculated the average intra-class correlation coefficient to measure agreement between experts.

### 2.3. Identification of Districts with Most Under-Reporting

To provide data to support the NTP’s programme planning, we identified the areas with most likely under-reporting according to the hackathon models. For this purpose, for each model we created maps displaying the ratio between new and relapse bacteriologically positive 2018 TB notification rate from NTP sources (numerator) and model predictions (denominator), by district. Low values of this ‘notifications to prevalence ratio’ for a particular district are assumed to correspond to the under-reporting of TB cases to and by the NTP. For each model, districts were allocated to a quantile based on this ratio and we identified those districts which consistently scored in the lowest quantile across all models.

## 3. Results

### 3.1. Comparison of Models

Details of the model building approaches can be found in Table 2 and Supplementary File S1. The hackathon models included a Bayesian binomial logistic regression with Markov Chain Monte Carlo (MCMC) inference (Model 1), an approximate Bayesian binomial logistic regression model with integrated nested Laplace approximations (INLA) inference (Model 2), an approximate Bayesian binomial-logistic model fit using the Broyden–Fletcher–Goldfarb–Shanno (BFGS) algorithm (Model 3), a Small Area Estimation and Latent Markov model with MCMC inference (Model 4) and artificial neural network followed by an Bayesian network (Model 5). In other words, all models were fitted within Bayesian inference frameworks; four were statistical models (Models 1–4) and one was a machine learning model (Model 5). Approaches to model selection included use of the log-scoring rule (Model 1), leave-one-out-cross-validation based on mean squared error and/or R^2^ (Models 2 and 3) and Chib’s estimator (Model 4) (see Supplementary File S1 for more details).

Details of variable selection strategies can be found in Table 2 and Supplementary File S1. By and large, the modelling teams made similar choices in terms of the outcome variable and candidate predictors, with varying choices in terms of data sources, geographical linkage and processing. All modelling teams used the raw TB prevalence provided by the NTP without performing multiple imputation of missing data or adjustment for non-responses. Model 1 stood out as the only model fitting cluster-level data disaggregated by age and sex. Model 2 and Model 3 both fitted TB cases at cluster-level whereas Model 4 and Model 5 fitted models at district-level. Four out of five models used TB notification data as candidate predictors, Model 3 being the only exception. Most models used non-TB health data as well as socio-demographic data. In addition, Model 3 used climate (precipitation) and civil unrest (protests and violent acts) data. Model 5 was the only model to use macroeconomic and development data (such as gross national income and human development index). Model 2 and Model 3 were the most granular models, including fine-scale predictor data (1 km or 5 km) for several health and socio-demographic indicators. Model 2 performed spatial kriging of predictors to obtain granular cluster-level predictors, with inter-survey estimates derived by linear interpolation between survey years; whereas Model 3 extracted point estimates of predictors from gridded data sources, when available, using the georeferenced survey cluster locations.

Final prediction models included between 7 and 10 variables (Table 2). Three out of five models (Models 1–3) did not include routine TB data in their final selected predictive models. While Model 3 did not include these in the list of potential covariates to start with, both Model 1 and Model 2 did not observe a significantly strong enough correlation between programmatic data (such as TB testing and notifications) and cluster-level TB prevalence rate and thus did not carry them forward into the model selection step (as has been documented elsewhere [2]). Overall, the models were fairly consistent in which local socio-demographic risk factors were associated with TB prevalence rates: poverty, underweight, urban extents, aridity and gender. Model 2 stands out as the only model with interactions.

### 3.2. Comparison of Predictions

All model predictions and accompanying credible intervals are provided in Supplementary File S2. Data quality appraisal statistics for all models are presented in Table 3, while prediction plots (histograms, precision plots and pairwise correlations) are presented in Supplementary Figures S1–S3.

Model 3 stands out as the model with the lowest district-wise mean and median estimates due to the calibration with Global Burden of Disease study 2017 national estimates [24] (Model 3: mean = 192/100,000, median = 162/100,000) while Model 1 stands out as the model with the highest mean and median estimates (Model 1: mean = 754/100,000, median = 378/100,000) (Supplementary Figure S1). Model 4 was the only model which predicted zero prevalence for a number of districts, with the 10th percentile equal to zero. The most complete set of predictions were provided by Model 2 (143 districts) and Model 3 (142 districts) whereas Model 1 provided estimates for 131 districts (due to linkage issues with certain covariate data sources) and Model 4 for 94 districts (those that had complete predictors for 2010–2011). According to the LOOCV R^2^ statistics, Model 3 scored the best in terms of pseudo-accuracy, defined as a model’s ability to predict cluster level TB in 2010–2011, when comparing with the actual measured values (R^2^ = 0.733), followed by Model 1 (R^2^ = 0.404), Model 2 (R^2^ = 0.320) and Model 5 (R^2^ = 0.115). Overall Model 2 and Model 4 provided the narrowest confidence intervals as can be seen by the lower precision ratios, whereas Model 3 has the widest confidence intervals. For Model 1 and Model 2, higher prevalence estimates tended to be less precise, while for Model 4 and Model 5, lower prevalence estimates tended to be less precise (Supplementary Figure S2). Within Model 3, no linear relationship was observed between the mean prevalence estimate for each district and the width of the uncertainty intervals surrounding that estimate.

**Figure 2:**
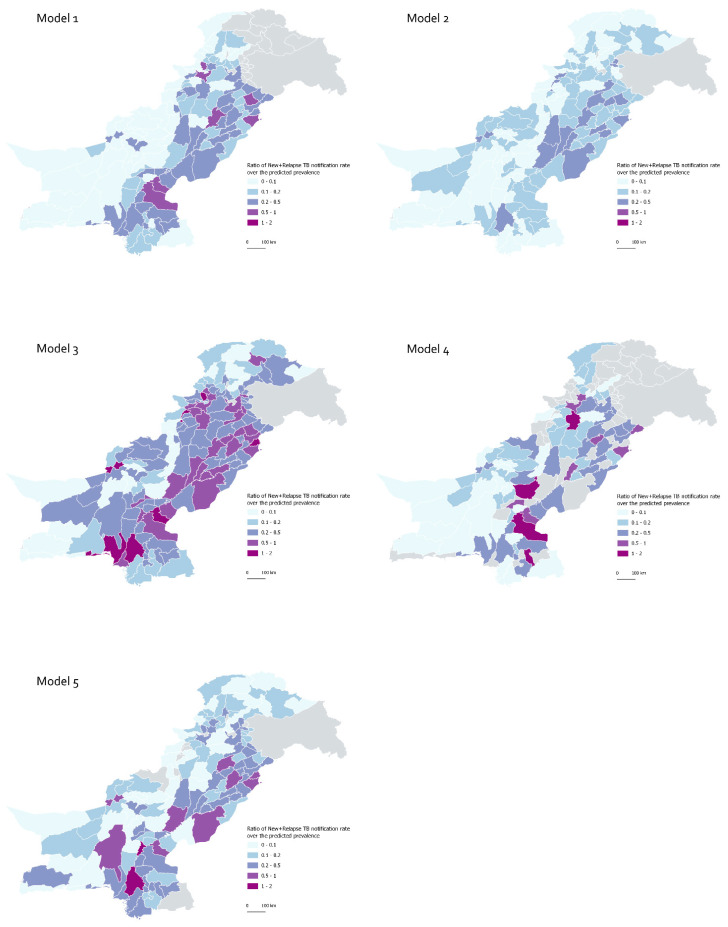
Predicted district-level TB prevalence in 2018, by model.

Overall there was very high heterogeneity in model predictions as can be seen from the five maps (Figure 2). Model 2 and Model 3 both present a more smoothed surface, which most likely reflects the high granularity of their approach as well as the spatially auto-correlated error structure used in the models. They are also the two most highly correlated estimates, by Pearson’s correlation coefficient, albeit weakly (r = 0.4029). Overall, Model 3 appears to be the most ‘average’ model, as it shares most similarities with other models (average pairwise correlations in district level central estimates of TB prevalence predictions) as can be seen in Table 3 and Figure 2 and Supplementary Figure S3. Model 3 and Model 5 obtained the highest average score (6.75) in the TB expert grading (Table 3). The average intra-class correlation coefficient between models was 0.92, indicating high clustering of grades within models (92% of the total variation in grades is between models) and thus high agreement between raters.

### 3.3. Identification of Districts with Most Under-Reporting

**Figure 3:**
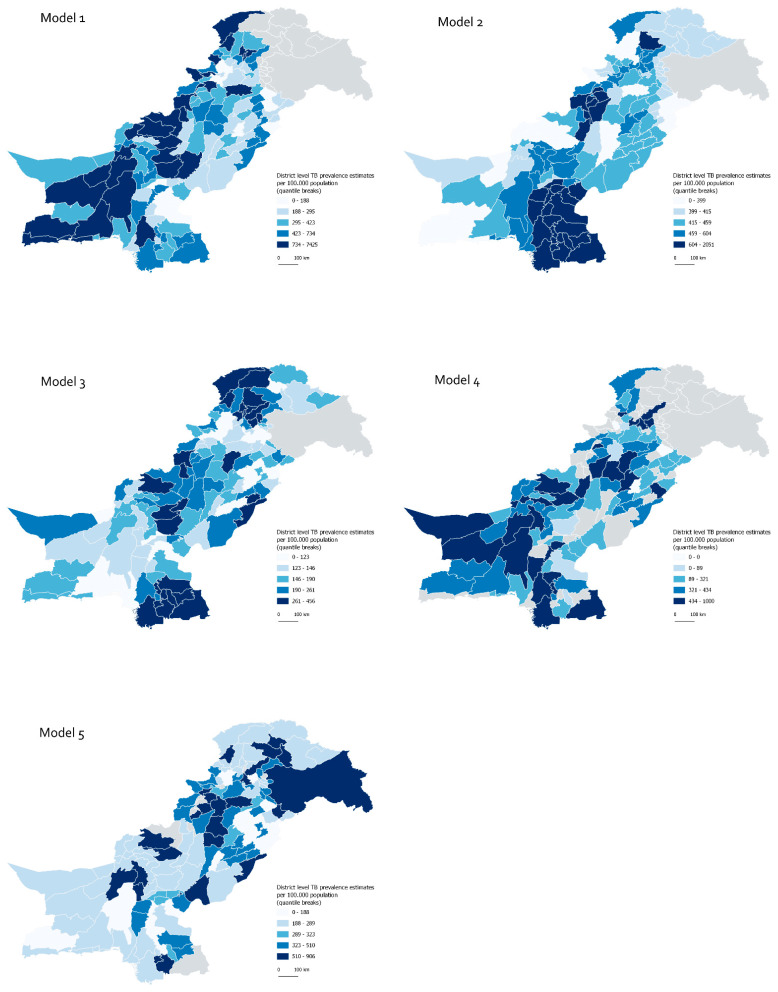
Ratio of 2018 new and relapse bacteriologically positive TB Notification rate over the predicted prevalence in each district, by model.

The maps displaying the ratio of 2018 new and relapse TB Notification rate over the predicted prevalence (Figure 3) provide information on areas with most under-reporting according to the model predictions. Areas consistently rated in the lowest notification rate to prevalence ratio quantile across all models include districts in Gilgit Baltistan in the north of the country (Ghizer and Ghanche districts); Khyber-Pakhtunkhwa in the north-west (Mohmand, Northern Waziristan, Southern Waziristan and Kurram) and Balochistan in the south-west (Kachhi, Musa Khel, Chagai, Kalat, Dera Bugti, Gwadar, Haranti and Lehri) (Supplementary Table S3).

## 4. Discussion

The TB hackathon provided five sub-national TB burden models that could be compared both in terms of their methodology and outputs. In doing so, the hackathon provided an opportunity to explore the utility of state-of-the-art modelling approaches to produce consistent TB prevalence predictions. It also proved useful to identify data sources which can be used to estimate TB prevalence at small spatial scales. The heterogeneity in model predictions shows that models based on a cross-sectional cluster-based prevalence surveys are limited in their ability to generate granular predictions into the future—even if they are complemented with other longitudinal and spatially disaggregated data sources. Indeed, model output comparisons highlighted the limited consensus between the different model outputs, and in the absence of an empirical ground truth against which to compare model predictions, it remains unclear which—and if any—predictive models produced reliable estimates. Nevertheless, we were able to identify a number of districts with consistently low notification to prevalence ratios across most models which could be prioritised for case finding activities.

Models 1–3 represent the more traditional statistical and epidemiological approach to TB modelling using a binomial logistic regression model. Although the predictions varied substantially across these three models, they shared a number of features. For instance, since these models were fitted using a logit link function, they could not generate estimates exactly equal to zero, and generally predicted values higher than the data in cases where prevalence estimates from the data are zero or relatively low. Furthermore, in Models 1 and 2, overall larger point estimates were associated with wider uncertainty. Models 4–5 changed the modelling perspective towards modern computational power and abundance of data. To the best of our knowledge, there are very few applications of Small Area Estimation and Latent Markov modelling (SAE-LM, Model 4) [25] and none on health data so far, and similarly there are limited examples of Bayesian Artificial Neural Network (ANN, Model 5) in health [26,27,28]. The main strength of these models lies in their flexibility, as there is no imposed function to link the outcome and predictors. This broadens options to the entire class of parametric probability distributions for the outcome. Both analytical and practical advantages follow. For instance, Model 4 was the only model able to estimate low district-level prevalence estimates and even zero. In contrast to Models 1 and 2, in Models 4 and 5 lower point estimates were associated with wider uncertainty.

Although we were not able to provide one set of validated estimates for sub-national TB planning, the NTP still found a number of practical applications for the hackathon models. The Pakistan NTP used Models 2 and 3 as a basis for sample size calculations for the upcoming TB prevalence survey (planned in 2022), given that they had both scored the highest as per the metrics presented in Table 3. Model 5, on the other hand, was used as a starting point to prioritise TB chest camps, a project the modellers became involved in shortly after participating in the hackathon. Chest camp data (including numbers of people screened, symptomatic, tested and positive) are now captured digitally in the field and are analysed in real-time. Incoming data are used to continuously update Model 5 predictions to provide ever more validated and accurate TB prevalence data at local level for the following decision making rounds on the location of chest-camps. Similarly, the NTP plans to use the hackathon models to operationalise other case-finding activities in their 2021–2023 National Strategic plan. In addition to chest camps, these include establishing sputum transportation mechanisms at primary health care levels, and engaging private providers in the diagnosis and treatment of TB. The hackathon data may be used, in combination with notification data, to understand the effectiveness of these interventions and further validate the accuracy of hackathon outputs.

One of the hackathon challenges was the combination of a spatial decomposition problem (from a national to district level estimates of TB) combined with a temporal forecast (projecting from 2010–2011 to 2018). While this was necessary to provide relevant information to NTP decision makers, it also added two layers of complexity for the modellers, each with their own theoretical and practical challenges. Spatial decomposition of survey data has known challenges, further compounded by the fact that TB is a rare and unevenly scattered attribute [1]. As a result, all models’ predictive power suffered from the following limitations: (1) data sparsity as a result of a limited number of clusters to base district-prevalence estimates (the 2010–2011 prevalence survey collected data from 95 [21] out of over 530 tehsils in the whole country [23]); (2) a small number of detected prevalent cases with over-dispersion in the distribution of clusters by case count (the mean number of bacteriologically positive cases was 3.3 per cluster with a standard deviation of 2.7 and 13/95 clusters with zero positive cases); and (3) extrapolation to areas where covariate data values fall outside the range observed in surveyed districts (the 95 survey clusters are in 68 unique districts whereas predictions were made for up to 136 districts). Moreover, both spatial and temporal forecasting rely on the strong assumption that the relationships observed in measured clusters and years (in our case 2010–2011) between TB estimates and covariates remain unchanged in non-measured clusters and future years (2018). However, there may be many violations to this assumption (migration patterns, consistency of quality of laboratory over time, lower TB bacteriology rate due to longer transport times, delays in transport, delays in testing, etc.).

The hackathon modellers also faced a number of more practical challenges. First of all, it is important to acknowledge that modellers were given a short turnaround and developed the models with limited human resources. Second, they faced a number of difficulties of working with the prevalence survey raw data, with limited information on data management steps (including imputation) needed beyond what was described in the survey report and scientific publication [20,21]. However, TB survey data are recognized as difficult to analyse, as there always are missing data and patterns of missingness are often associated with the outcome of interest (prevalence). WHO provides ample guidance on strategies to deal with these issues [29], but the specific choices of the Pakistan TB prevalence survey analysts were not available to the hackathon modellers. This underscores the importance of implementing TB prevalence surveys with transparent and reproducible procedures for data management and data analyses as recommended by most good epidemiological practice guidelines, including recently developed guidelines specifically for global health [30]. All underlying programming code and data for the hackathon models are available on an open access Zenodo repository (see Data Availability Statement). We invite interested modellers to access the code and data to improve on our estimates—either with methodological advancements or by including new high-quality predictors of TB prevalence.

## 5. Conclusions

The TB hackathon provided a unique opportunity to compare different TB subnational prediction models, including novel modelling techniques which had never been applied in this domain before. The technical difficulty of the hackathon assignment highlighted the known challenges of satisfying stakeholder information needs (most recent district-wise estimates) while attempting to fit complex statistical methodologies (subnational decomposition and temporal forecast). The large heterogeneity between the various outputs serves as an important note of caution for the future production and use of granular predictive models of TB based on cross-sectional cluster-based prevalence survey data. Nevertheless, by soliciting and contrasting different methodologies for this challenging problem, the hackathon was successful in providing examples of a range of applications for modellers interested in further developing or refining their approaches to subnational disease modelling. Moreover, the NTP’s use and plans for these outputs shows that, limitations notwithstanding, they are valued by decision makers and planners.

## Figures and Tables

**Table 1:** Datasets made available to TB hackathon modelers by Pakistan NTP.

Dataset	Disaggregation	Time Period
1. Prevalence survey data ^1^	Individual	2010–2011
2. TB notifications	District	quarterly 2009–2018
3. Laboratory External Quality Assessment data	District	quarterly 2013–2017
4. Drug-sensitive TB treatment outcomes data	District	quarterly 2009–2015
5. Drug-Resistant TB notifications	District	quarterly 2009–2018
6. Master list of TB facilities	Health facility	2019
7. Sputum smear testing data	District	quarterly 2009–2017
8. Private sector notifications	District	Yearly 2017–2018
9. HIV registrations	Province	2001–2018
10. HIV testing rates among TB cases	District	quarterly 2009–2018
11. Census Population estimates	District	2017
12. Shape files	District	2019

^1^ Including village names corresponding to the survey-clusters

**Table 2:** Model specifications.

	Model 1	Model 2	Model 3	Model 4	Model 5
**Modelling framework**	Binomial-logistic regression	Binomial-logistic regression	Binomial-logistic regression	Small Area Estimation (SAE) and Latent Markov (LM) modelling as linking model for SAE	Self-Organising Maps (SOM) on binomial
**Inference**	Bayesian inference with Markov Chain Monte Carlo with No-U-Turn-Sampler (NUTS)	Approximate Bayesian inference with integrated nested Laplace Approximations (INLA)	Approximate Bayesian inference with Broyden–Fletcher–Goldfarb–Shanno algorithm	Bayesian inference with Data Augmentation Markov Chain Monte Carlo and Gibbs sampler	Bayesian Artificial Neural Network
**Covariance structure**	Spatially explicit hierarchical model with fixed and random effects.	Spatially explicit hierarchical model with fixed and random effects	Spatially explicit hierarchical model with fixed and random effects	Hierarchical Discrete latent state model depending on a Gaussian linking model	N/A
**Outcome variable**	Bacteriologically-confirmed TB cases from TB prevalence survey at cluster-level by age and sex	Bacteriologically-confirmed TB cases from TB prevalence survey at cluster-level	Bacteriologically-confirmed TB cases from TB prevalence survey at cluster-level	Bacteriologically-confirmed TB cases from TB prevalence survey at district level	Bacteriologically-confirmed TB cases from TB prevalence survey at district level
**Final set of predictors ^1^**	SES, HH size, Indoor smoke, BMI, WAZ, Vaccination coverage, Prevalence of cough, Distance to health facility	Age 15–24 Female Age 15–24 * female Ag 65+ Age_65+ * Sindh Underweight Underweight * KPH	Population density Access to cities [10] Density of TB facilities Poverty Urban extents Locations of protests Locations of violent acts Aridity	Urban households Rural households Urban male pop Rural male pop Urban female pop Rural female pop Pop growth overall Bac+ notifications Bac- notifications EP notifications	All-forms TB notifications Bac+ TB notifications SS+ rate among tested Population density Average household size Percentage rural population Growth rate (urban, rural) Sex ratio (urban, rural) Log gross national income Life expectancy Expected years of schooling Mean years of schooling Human development index

^1^ x1: x2 represents factor multiplication, while x1 * x2 represents factor crossing and is equivalent to x1 + x2 + x1: x2.

**Table 3:** Predictions: data quality appraisal.

	Model 1	Model 2	Model 3	Model 4	Model 5
**Summary statistics ^1^**	Min = 104 Max = 7425 Mean = 754 Median = 378	Min = 276 Max = 2050 Mean = 508 Median = 430	Min = 51 Max = 456 Mean = 192 Median = 162	Min = 0 Max = 1000 Mean = 362 Median = 382	Min = 44 Max = 906 Mean = 366 Median = 289
**Completenes ^2^**	131	143	142	94	139
**Pseudo-accuracy by LOOCV for 2010 ^3^**	R^2^ = 0.404	R^2^ = 0.320	R^2^ = 0.733 ^4^		R^2^ = 0.115
**Cross-validation ^5^**	Model 2: r = −0.0882 Model 3: r = 0.2305 Model 4: r = −0.0041 Model 5: r = 0.0001	Model 1: r = −0.0882 Model 3: r = 0.4029 Model 4: r = 0.2492 Model 5: r = 0.1495	Model 1: r = 0.2305 Model 2: r = 0.4029 Model 4: r = 0.2402 Model 5: r = 0.1583	Model 1: r = −0.0041 Model 2: r = 0.2492 Model 3: r = 0.2402 Model 5: r = 0.0778	Model 1: r = 0.0001 Model 2: r = 0.1495 Model 3: r = 0.1583 Model 4: r = 0.0778
**Precision ^6^**	Ratio = 2.69	Ratio = 0.78	Ratio = 5.30	Ratio = 0.63	Ratio = 2.06
**Credibility score ^7^**	Rater 1: 3 Rater 2: 4 Rater 3: 3 Rater 4: 5 Mean score = 3.75	Rater 1: 5 Rater 2: 3 Rater 3: 3 Rater 4: 5 Mean score = 4	Rater 1: 7 Rater 2: 7 Rater 3: 7 Rater 4: 6 Mean score = 6.75	Rater 1: 4 Rater 2: 4 Rater 3: 3 Rater 4: 5 Mean score = 4	Rater 1: 7 Rater 2: 8 Rater 3: 6 Rater 4: 6 Mean score = 6.75

^1^ Prevalence per 100,000 inhabitants. ^2^ Out of 143 districts. The difference between 136 and 143 is accounted for by districts in contested areas of Pakistan: 1 district in India-administered Kashmir, 1 district in Pakistan-administered Kashmir, 3 districts in the Federally Administered Tribal Area (FATA) and 2 districts in Balochistan. ^3^ LOOCV comparing final model estimates for 2010–2011 with actual prevalence survey cluster-level estimates for 2010–2011. This could not be calculated for Model 4 as LOOCV metrics are not practical for SAE-LM models (computationally too intensive) and were not produced by Model 5. ^4^ When performing cross validation, Model 3 excluded each cluster from the original survey; this meant that for cluster observations that were originally geo-matched to admin3 units and then resampled to multiple admin4 centroids, all down-sampled points corresponding to a single survey observation were excluded from a single out-of-sample run. ^5^ Pairwise correlations of district level central estimates of TB prevalence predictions (Pearson’s correlation coefficient). ^6^ Ratio = [(upper limit of 95% credible interval) − (lower limit of 95% credible interval)]/(prevalence estimate). ^7^ Four TB experts from the Pakistan National TB control Programme were asked to grade models from 1–10 based on how credible they deemed the model estimates.

## Data Availability

All underlying programming code and data for the hackathon models are available in an open access Zenodo repository (zenodo.org, DOI: http://doi.org/10.5281/zenodo.5112022).

## Supplementary Materials

The following supporting information can be downloaded at: https://www.mdpi.com/article/10.3390/tropicalmed7010013/s1, Table S1: Additional model specifications, by model; Table S2: Model predictions, by district and model; Table S3: Notification to prevalence, by district and model; Figure S1: Histograms of model predictions, by model; Figure S2: Precision vs. point estimate, by model; Figure S3: Pairwise correlations between model predictions.
