# Supplementary material for: TB Hackathon: Development and Comparison of Five Models to Predict Subnational Tuberculosis Prevalence in Pakistan
Source: Trop Med Infect Dis. 2022 Jan 17;7(1):13. doi: 10.3390/tropicalmed7010013 (PMC8780063; doi:10.3390/tropicalmed7010013)
Supplement: Supplementary file 1 [file tropicalmed-07-00013-s001.zip › tropicalmed-1417964-supplementary.pdf]

Tropical Medicine and Infectious Disease

Supplementary material of TB Hackathon: Development and Comparison of Five Models to Predict Subnational Tuberculosis Prevalence in Pakistan

Table S1. Additional model specifications.

	Model 1	Model 2	Model 3	Model 4	Model 5
Candidate predictors	TB (district) [1]:<br>• Age-and sex-category specific bac+ notifications 2009-2012<br><br>Health (district estimates derived from cluster-level data) [2]:<br>• body mass index<br>• weight-for-age Z-score<br>• prevalence of vaccination<br>• prevalence of BCG<br>• prevalence of chronic cough<br>• self-reported TB prevalence<br>• distance to nearest health facility<br>• awareness of TB<br><br>Socio-demographic (district) [2]:<br>• mean household size<br>• SES wealth score<br>• indoor smoke, smoking	TB (district) [1]:<br>• Bac+ notifications<br>• All forms notifications<br><br>Health (cluster):<br>• health visits in last 12 month [2]<br><br>Socio-demographic data<br>• Solid cooking fuel (cluster) [2]<br>• Lowest wealth quintile (cluster) [2]<br>• Rural classification (cluster) [2]<br>• Underweight [2] (cluster)<br>• Multidimensional poverty index 2007 (1km) [3]<br>• Built settlement growth model (100m) [3]<br>• Settlement type, (1km) [4]	Health<br>• Number of facilities in district per 100,000 [1]<br>• Population density: number of persons per 1km_sq [3]<br>• Time required to travel to nearest settlement by surface travel at 5x5 km [3]<br><br>Socio-demographic (district)<br>• People per 1km_sq living in poverty in 2007 [5]<br>• Urban locations<br>• Protests per year [5]<br>• Violent acts per year [5]<br><br>Climate [7]:<br>• Mean annual precipitation/ evapo-transpiration 1950 – 2000. (1km resolution)	TB (district) [1]:<br>• Bac+ notifications 2011-16<br>• Bac- notifications 2011-16<br>• EP notifications 2011-16<br>• All forms notified 2017<br>• Total slides tested 2011-16<br>• Slide errors 2011-16<br><br>Health [1]<br>• Notified HIV total 2011-18<br>• Facilities total 2013-16<br><br>Socio-demographic (district) [7]<br>• Households total 2017 (total, urban, rural)<br>• Population total 2017 by gender (total, urban, rural)<br>• Sex ratio 2017 (total, urban, rural)<br>• Growth rate 2017 (total, urban, rural)	TB (district) [1]:<br>• All-forms TB notifications<br>• Bac+ TB notifications<br>• SS+ rate among tested<br><br>Socio-demographic (district/province)<br>• Population density [6]<br>• Average household size [6]<br>• Percentage rural population [6]<br>• Growth rate (urban, rural) [6]<br>• Sex ratio (urban, rural) [6]<br>• Log gross national income [3]<br>• Life expectancy [3]<br>• Expected years of schooling [3]<br>• Mean years of schooling [3]<br>• Human development index [8]
Processing of predictors		Linear combinations. Interaction terms Spatial kriging to obtain cluster-level estimates. Inter-survey values derived by linear interpolation	Covariate values truncated to minimum and maximum values observed in the model fit.	Covariates used individually at district level. At cluster level the same value of covariates was assigned to all clusters included in that district	Data reduction of spatially explicit climatic variables using Self Organising Maps
Lowest level of spatial aggregation	District	Cluster	Cluster	District	District
Model Selection	Log-scoring rule (expected value of −log (pr(observation) when predicting the probability of sampled individuals in a district having TB based data only in other districts), as well as MSE and RSE. Visual inspection of diagnostic plots also informed final choice of model	Covariates with correlation coefficient p<0.2 considered for use in the subsequent model selection step. Various models specified including different sets of covariates. For each model, used leave-one-out cross-validation on MSE.	Initial list of candidate covariates developed based on known or postulated relationships with TB. Variance inflation factor (VIF) analysis used to reduce multicollinearity. Candidate models created using 4 VIF thresholds. Final model selected through leave-one-out cross validation on MSE and R_sq	Best model selected by means of Chib's estimator and the Bayesian Information Criterion. Highly correlated variables detected and reduced. Step-wise regression to identify sub-sets of relevant covariates. Model fitting completed by grid search to select latent model main parameter value	Single entry of environmental variables used in a SOM which iteratively mined for latent factors. 25 resulting node representations (maps) included in a district level Bayesian network to detect patterns in the district level environmental, socio-economic and TB programmatic data.

Predictions	To maintain consistency with published estimates, raw central estimates and uncertainty were scaled to match. Per capita results were projected using the WHO estimated trend (and uncertainty) and updated district sizes. These were aggregated over sex/age	Model was refit on the entire prevalence survey data set and covariate data set from 2011, resulting in 1000 parameter draws for the set of coefficients. Coefficients were applied to the raster-level data for the entire country for 2018 aggregated at a 5-km resolution.	Generated initial estimates at the 5x 5km-grid cell to align with resolution available for existing covariates and subsequently constructed estimates at the district level using the values of each grid cell weighted by the underlying population raster. Calibrated national-level prevalence estimates to results from GBD 2017 (22)	Primary outputs are district-wise TB prevalence estimates for every year in the period 2011-2016. Estimated initial probabilities give overall average probability, for a given district, to be classified in one of three increasing levels of TB burden in 2011. A matrix of transition probabilities predicts each district's chance of changing class in 2018 (improve or worsen)	Model was trained using known district human development index (HDI) for the year 2011-2015 and then to predict district-wise HDI for 2016, 2017 and 2018. Model predictions fitted to district level TB prevalence rates.

Data sources: [1] Pakistan National TB Control Program; [2] Pakistan Demographic and Health Survey 2017-18 (30); [3] worldpop.org; [4] European Commission Global Health Settlement; [5] Humanitarian Data Exchange (31); [6] Pakistan 2017 census (20); [7] Zomer et al (32); [8] Global Data Lab(33).

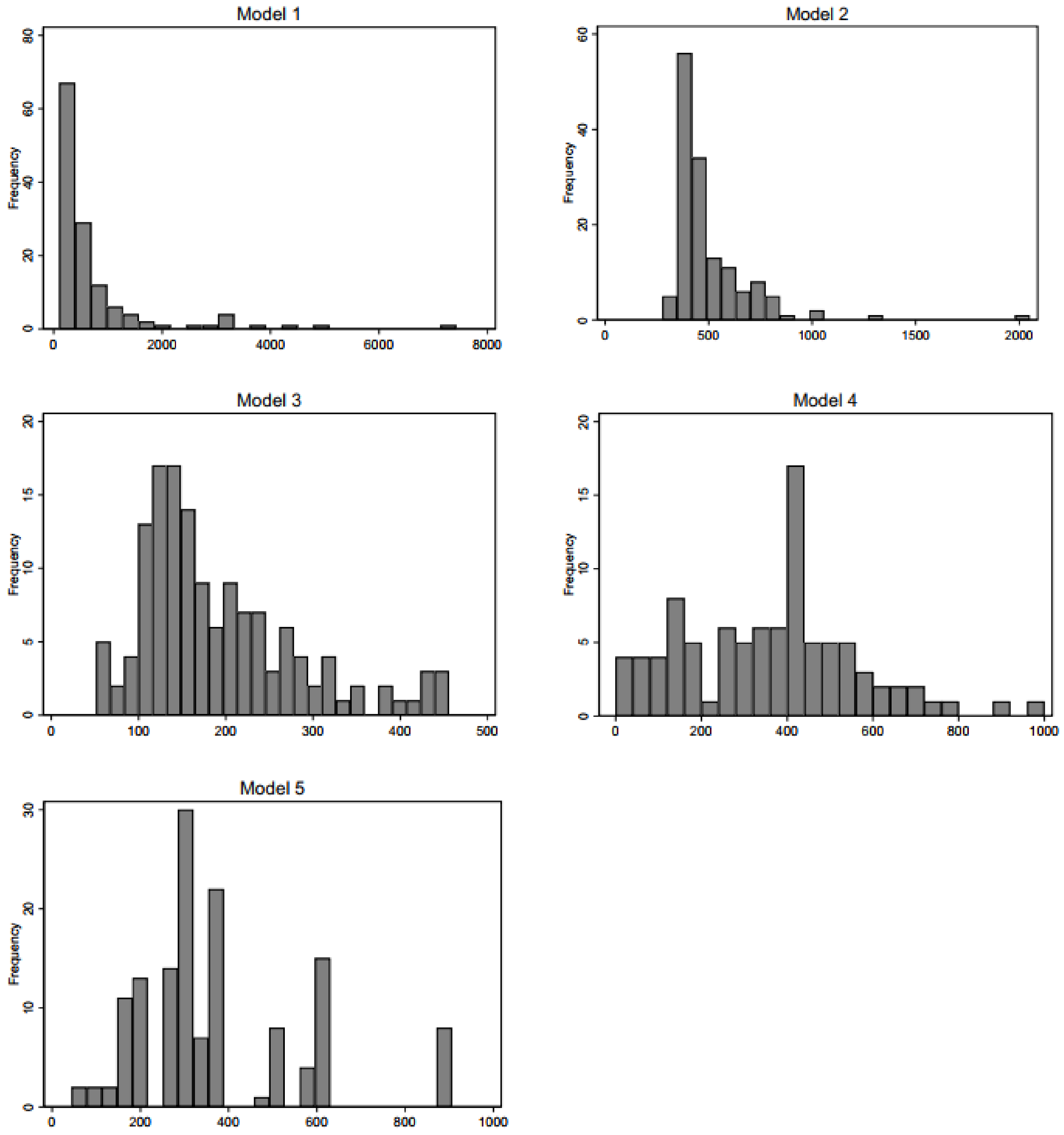

Figure S1. Histograms of model predictions, by model.

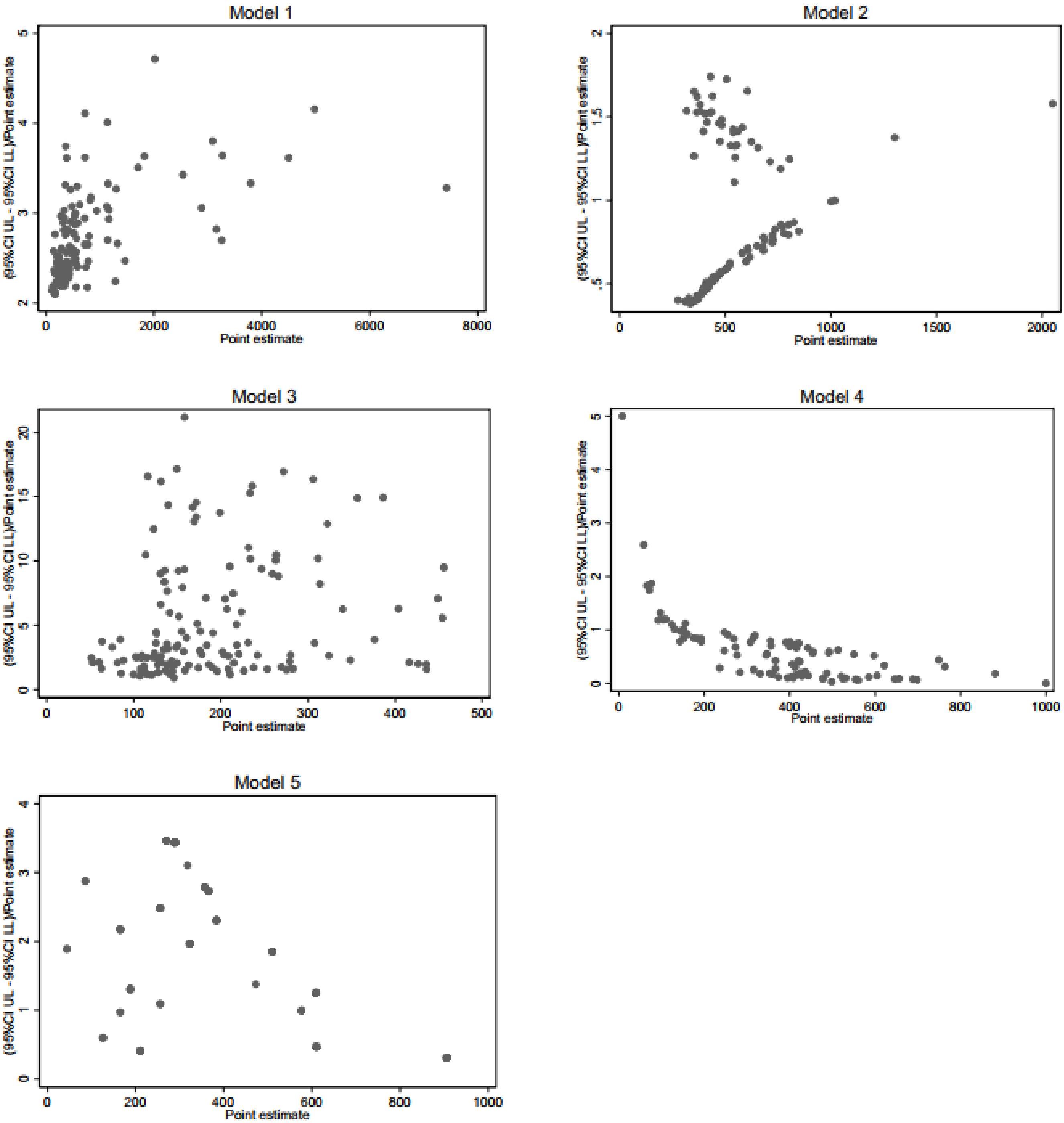

Figure S2. Precision vs. point estimate, by model.

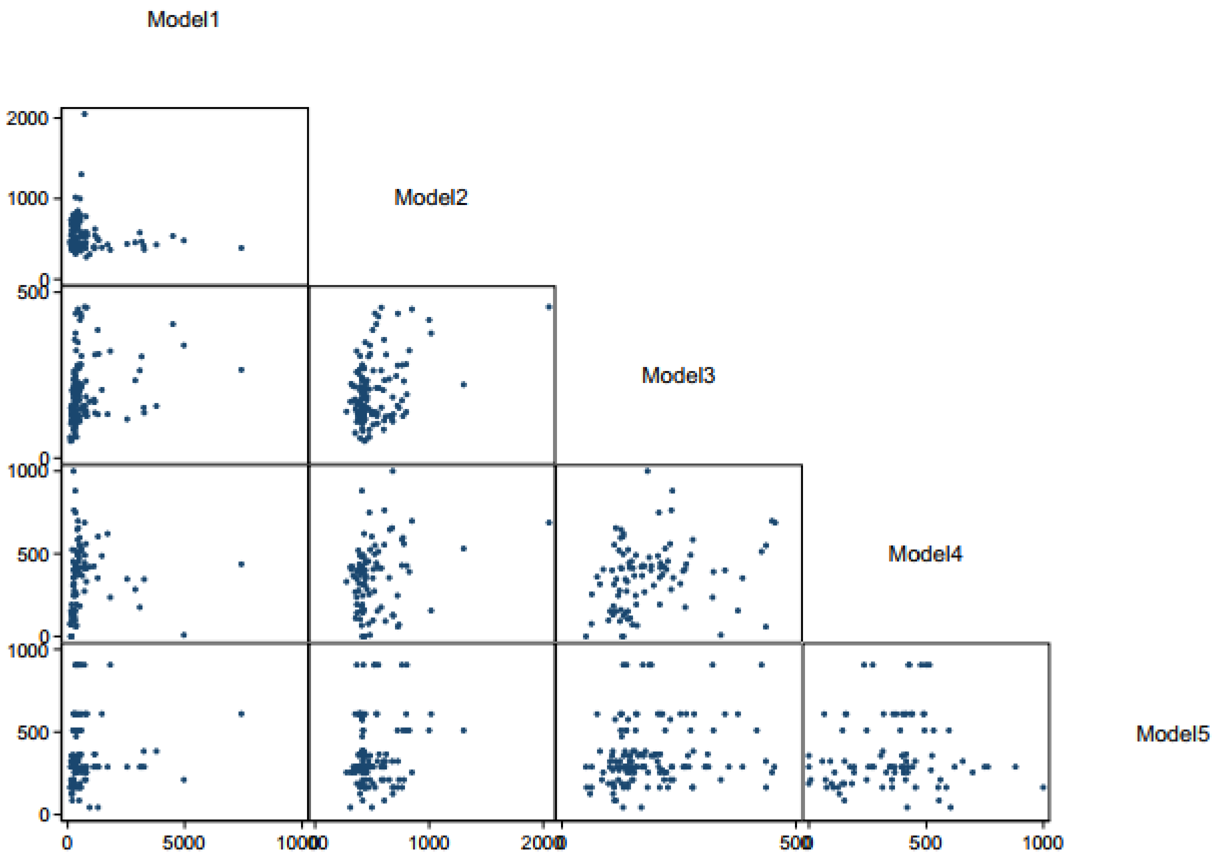

Figure S3. Pairwise correlations between model predictions.
